# Synthesis and potent cytotoxic activity of a novel diosgenin derivative and its phytosomes against lung cancer cells

**DOI:** 10.3762/bjnano.10.189

**Published:** 2019-09-24

**Authors:** Liang Xu, Dekang Xu, Ziying Li, Yu Gao, Haijun Chen

**Affiliations:** 1 Cancer Metastasis Alert and Prevention Center, College of Chemistry, Fuzhou University, Fuzhou, Fujian 350116, China; 2 Fujian Provincial Key Laboratory of Cancer Metastasis Chemoprevention and Chemotherapy, Fuzhou University, Fuzhou, Fujian 350116, China; 3 College of Chemistry, Fuzhou University, Fuzhou, Fujian 350108, China

**Keywords:** diosgenin, non-small-cell lung cancer, phytosomes, sterol structure

## Abstract

Diosgenin (Di), a steroidal sapogenin derived from plants, has been shown to exert anticancer effects in preclinical studies. Using Di as a starting material, various Di derivatives were designed and synthesized, aiming to discover new steroid-based antitumor agents. In this work, we synthesized several Di derivatives and screened FZU-0021-194-P2 (P2), which showed more potent cytotoxic activities against human non-small-cell lung cancer A549 and PC9 cells. Considering that Di has a unique sterol structure similarly to cholesterol, P2 phytosomes (P2Ps) were prepared to further improve the water solubility of P2. The P2Ps exhibited a particle size of 53.6 ± 0.3 nm with oval shape and a zeta potential of −4.0 ± 0.7 mV. P2Ps could inhibit the proliferation of lung cancer cells more efficiently than Di phytosomes after 72 h of incubation time by inducing cell cycle arrest and apoptosis. The results indicated that P2Ps could be a promising anticancer formulation for non-small-cell lung cancer.

## Introduction

Natural products are the most easily accessible source of lead compounds of anticancer drugs [1]. In recent years, chemists have been seeking extensively for effective new chemical entities from natural products and their derivatives. Diosgenin (3β-hydroxy-5-spirostene, Di), is a conventional herbal sterol distilled from yams, fenugreek, and *Cheilocostus speciosus* [2]. Prominently, it is a characteristic initial intermediate for the synthesis of steroidal compositions, oral contraceptives and sex hormones. A series of preclinical and mechanistic investigations showed that Di contributed to multiple anticancer activities, such as restraining the hTERT gene expression in A549 lung cancer cells [3], inhibiting breast cancer stem-like cells via Wnt β-catenin signaling [4], impeding hepatocellular carcinoma cells by increasing DDX3 expression [5], and inducing apoptosis of prostate cancer cells through activation of estrogen receptor-β [6]. Additionally, studies demonstrated that Di has unique preventive/therapeutic outcomes not only against tumors, but also for other diseases such as diabetes [7], myocardial infarction (AMI) [8], acute liver injury [9], goiter [10], and Alzheimer’s disease (AD) [11].

In consideration of the diverse biological activities of Di, researchers are interested in modifying the steroid structure of Di to obtain Di derivatives that have more efficient anticancer activities. 1-Phenyl-(1*H*-1,2,3-triazol-4-yl)methoxy diosgenin showed an IC_50_ value against A549 cells that was about a third of that of Di [12]. Diosgenin–imidazolium salt derivatives were also synthesized and displayed significant cytotoxic activities against several human cancer cell lines [13]. One of novel 22-oxo-26-selenocyanocholestanic steroids based on Di synthesized by Fernández-Herrera et al. exhibited remarkable antiproliferative activity against HeLa cells, which is close to that of the clinical anticancer agent paclitaxel [14]. These successful examples demonstrated that the steroidal structure of Di is promising for the discovery of new anticancer drugs.

Liposomes that usually consist of phospholipids and are stabilized by cholesterol have been substantially investigated as drug carriers for targeting, modulating drug pharmacokinetics, and decreasing drug toxicity [15–16]. Liposomes also can be used as solubilizing media to enhance solubility and bioavailability of insoluble drugs [17]. Di and our prepared analogues have a sterol structure that is very similar to cholesterol. It was reported that Di and lipids formed highly stable complexes at the air–water interface [18]. Therefore, Di and its derivatives could be substituted for cholesterol to form novel stable liposome-like phytosomes. The prepared phytosomes were expected to enhance the solubility and bioavailability of the Di derivative for clinical transformation.

In this study, we first prepared several Di derivatives and screened the most potent candidate through a preliminary structure–activity relationship study. Then the liposome-like phytosomes were prepared by substituting Di and its derivative for cholesterol. The anticancer effects of free drugs and their phytosomes were investigated in non-small-cell lung cancer cells.

## Results and Discussion

### Synthesis and characterization of Di derivatives

**Figure 1:**
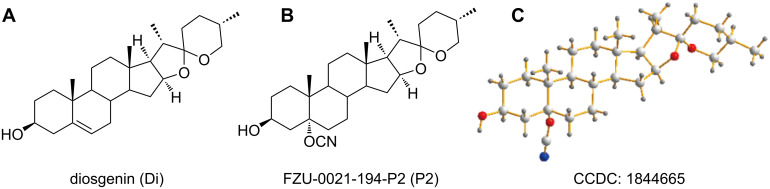
(A) The structure of diosgenin. (B) The structure of FZU-0021-194-P2. (C) X-ray crystallographic analysis of FZU-0021-194-P2. CCDC 1844665 contains the crystallographic data of P2. The data can be obtained free of charge from the Cambridge Crystallographic Data Center.

Late-stage functionalization that uses the C–H bonds as points of potential variations is a useful method to generate novel analogues of a lead structure without depending on de novo synthesis [19]. It is widely accepted that the oxidation of aliphatic C–H bonds could be an efficient approach to diversify complex structures [20]. In the steroid structure of Di, there are abundant electrons at double-bond sites for electrophilic addition. Based on this feature, we designed and synthesized a series of Di derivatives. We investigated the cytotoxicity of these Di derivatives in different cancer cell lines and their IC_50_ values were calculated. The novel Di derivative FZU-0021-194-P2 (P2) showed the most efficient suppression of cell growth (Figure 1). P2 was characterized by ^1^H NMR (Supporting Information File 1, Figure S1), ^13^C NMR (Supporting Information File 1, Figure S2) and HRMS. The structure of P2 was further established by X-ray crystallographic analysis. CCDC1844665 contains the crystallographic data of P2. The data can be obtained free of charge from the Cambridge Crystallographic Data Center.

### Antiproliferative activity of P2

**Figure 2:**
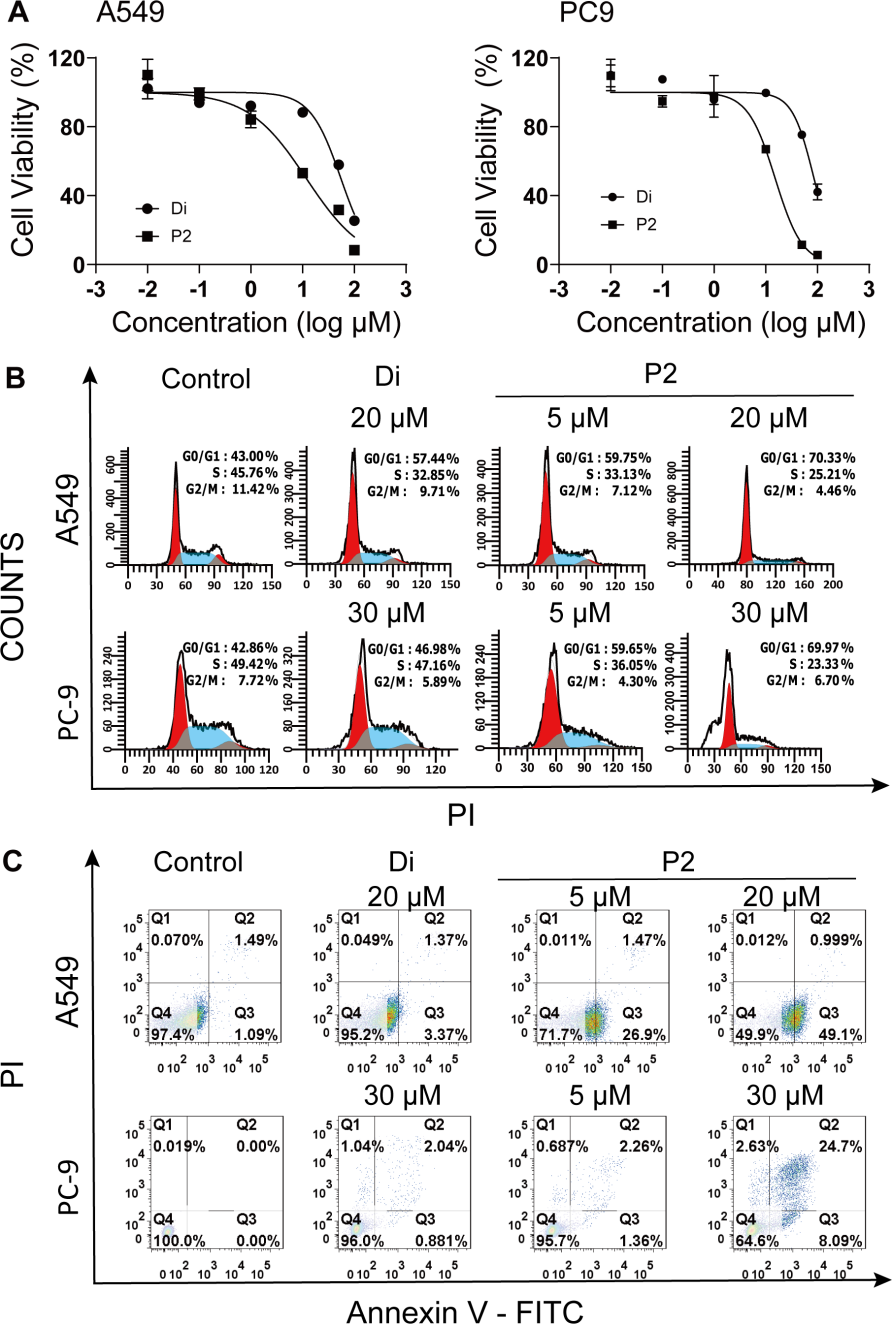
In vitro anticancer activity of diosgenin (Di) and its derivative FZU-0021-194-P2 (P2). (A) The antiproliferative activities of different concentrations of Di and P2 against A549 and PC9 cells for 48 h. (B) Cell cycle analysis of A549 and PC9 cells after treated with Di and P2 for 48 h. (C) Cell apoptosis analysis of A549 and PC9 cells after treated with Di and P2 for 48 h.

The anticancer activity of Di and its analogue P2 was tested in human lung cancer cell lines A549 and PC9, human cervical cancer HeLa cell line, and human hepatoma HepG2 cell line using MTT assays. All cells were treated with each compound at the indicated concentrations for 48 h. It was found that Di and P2 suppressed two lung cancer cells better than HeLa and HepG2 cells (Table 1). The IC_50_ values of P2 in A549 and PC9 cells were 11.8 and 15.2 μM, respectively, which were much lower than those of Di (55.0 and 85.8 μM, respectively). Compared with the parent drug, the construction of quaternary carbon centers in the derivative might endow P2 with a better flexibility to enter cells more easily. The cyano group in P2 might increase the binding capability of P2 to specific proteins to improve its anticancer potency. Figure 2A shows that Di and P2 could suppress the cell viability in a dosage-dependent manner in A549 and PC9 cells. All the results demonstrated that P2 had much stronger antiproliferative activity against A549 and PC9 cells than Di.

**Table 1:** The IC_50_ values of diosgenin (Di), FZU-0021-194-P2 (P2) and their phytosomes against cancer cells by MTT assay.

no.	sample	IC_50_/μM			

		A549	PC9	HeLa	HepG2

1	Di	55.0 (48 h)	85.8 (48 h)	103.6 (48 h)	410.9 (48 h)
		39.9 (72 h)	51.7 (72 h)	—	—
2	P2	11.8 (48 h)	15.2 (48 h)	54.7 (48 h)	40.4 (48 h)
		5.1 (72 h)	8.7 (72 h)	—	—
3	DiP^a^	18.0 (72 h)	29.1 (72 h)	—	—
4	P2P^b^	8.3 (72 h)	8.2 (72 h)	—	—

^a^DiP: diosgenin phytosomes; ^b^P2P: FZU-0021-194-P2 phytosomes.

### Cell cycle arrest and apoptosis induced by P2

The cell cycle is the series of events that take place in cells for their propagation and multiplication. The phases consist of the gap-1 phase (G1), synthesis phase (S), mitosis phase (M), and gap-2 phase (G2). Di induced cell cycle arrest in different phases in different cancer cells. It was reported that Di could induce G2/M cell cycle arrest in liver cancer cells [21], arrest SCC cells at the sub-G1 phase [22], and impede cell cycle progression in the G0/G1 phase in A549 cells [23]. To determine the antiproliferative mechanisms of Di and P2, the effects of Di and P2 on cell cycle distributions were examined in A549 and PC9 cells. As shown in Figure 2B, Di and P2 could induce A549 and PC9 cell cycle arrest in G0/G1 phase (at a concentration 20 μM for A549 cells and 30 μM for PC9 cells for 48 h). To our surprise, the cell proportion in G0/G1 phase induced by P2 at a low concentration (5 μM) was exceeding that of Di at high concentrations. These results demonstrated that P2 was more potent than Di to inhibit cell cycle progression.

Apoptosis, the programmed cell death, is a physiological process that is accurately regulated at genetic level resulting in the removal of damaged cells in a controlled manner [24]. Most of the chemotherapeutic drugs exert effects through the mechanism of apoptosis. It was reported that diosgenin could induce early apoptosis in human prostate DU145 cancer cells [25] and cause late apoptosis in human cervical HeLa cancer cells [26]. In order to explore the death mechanism of P2, A549 and PC9 cells were treated with P2 and Di for 48 h and the proportion of cells undergone apoptosis was counted through Annexin V-FITC/PI staining and followed by flow cytometry analysis. As shown in Figure 2C, each scatter plot is divided into four quadrants. The cells in Q1, Q2, Q3, and Q4 quadrants represent necrotic cells, late apoptotic cells, early apoptotic cells, and viable cells, respectively. The results showed that P2 significantly induced apoptosis in both A549 and PC9 cells compared with Di. In A549 cells, P2 at 20 μM induced 51.1% early apoptosis, while Di at the same concentration induced only 3.75% early apoptosis. P2 at 5 μM showed even stronger early apoptosis induction effects than Di at 20 μM. In PC9 cells, P2 at 30 μM induced 34.4% early and late apoptosis, while Di only induced 6.7% early and late apoptosis. The results clearly showed that P2 was more potent than Di in inducing apoptosis in A549 and PC9 cells.

### Preparation and characterization of P2 phytosomes (P2P)

Formulating small-molecule drugs into nanometer-scaled particles have been widely investigated for targeted drug delivery to tumors. These nanoparticles can passively accumulate in tumors via enhanced permeability and retention (EPR) effect, thus decreasing the toxicity of nonselective bio-distribution [27]. Considering the advantages of liposomes as a drug delivery system for chemotherapeutic drugs and the similar sterol structures in cholesterol and Di, complexes of phospholipids and Di or P2 were prepared to further enhance the solubility and improve the pharmacokinetic profiles of Di and P2 for better clinical transformation.

Phytosomes are lipid-compatible molecular complexes of phospholipids and natural active ingredients in which the active ingredients, which contain sufficient polar functional groups such as COOH, OH, and NH_2_, are anchored to the polar head of phospholipids by polar and hydrogen-bond interactions [28]. Because of the interactions, phospholipids and natural active ingredients could undergo self-assembly into stable vesicles in aqueous solution, which could act as a vehicle to facilitate membrane transport [29]. Compared to liposomes, phytosomes can load more drug molecules, and showed enhanced stability in the lyophilization and reconstitution processes prior to use [30]. Phytosomes have been used as drug delivery systems of several insoluble natural drugs in recent years. Sinigrin [31] and epigallocatechin-3-*O*-gallate [32] loaded in phytosomes showed stronger antiproliferative activity than free drugs against melanoma cells and breast cancer cells.

**Figure 3:**
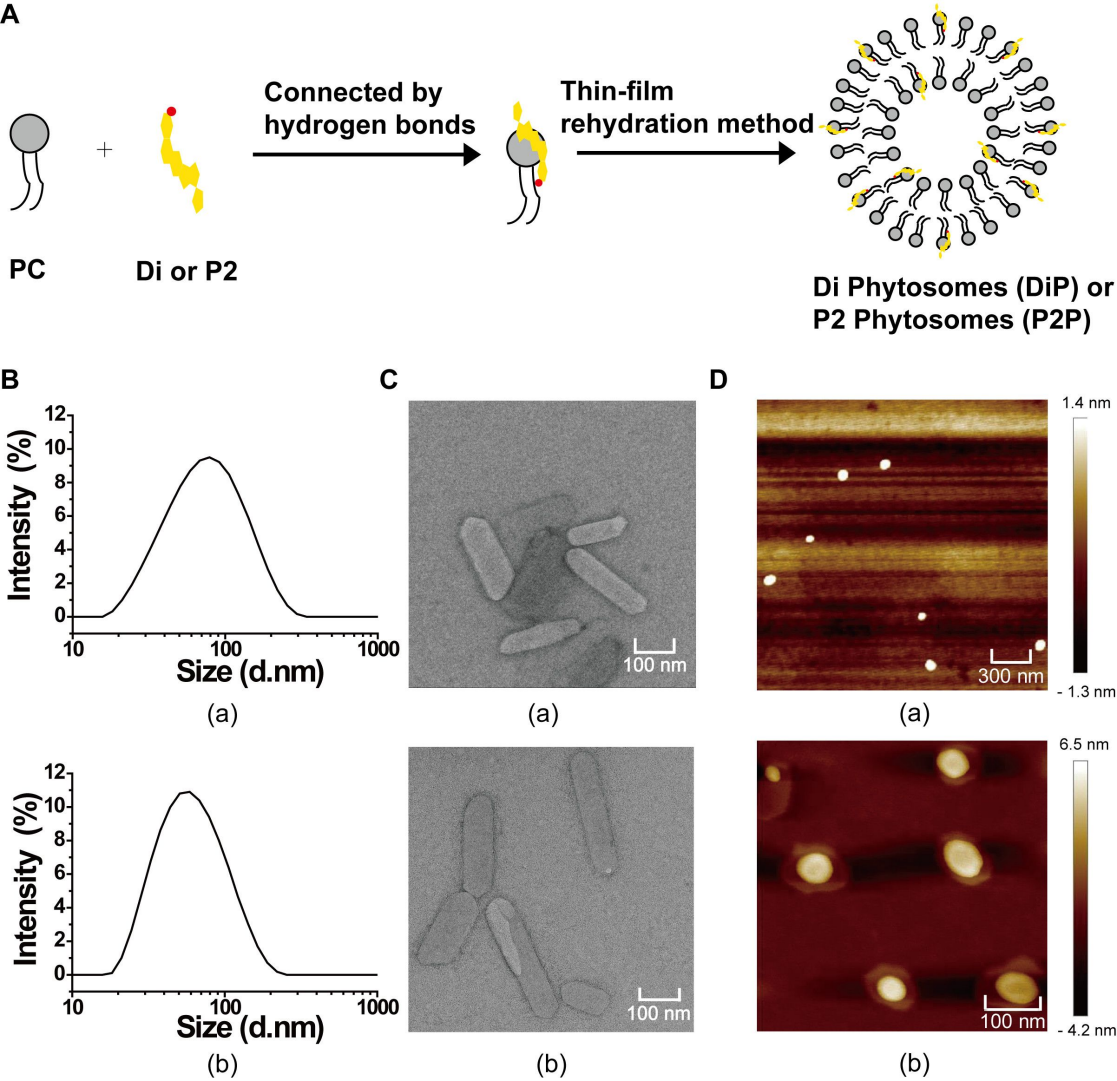
Preparation and characterization of Di phytosomes (DiP) and P2 phytosomes (P2P). (A) Schematic illustration of the preparation processes for DiP and P2P. (B) DLS measurements of DiP (a) and P2P (b). (C) TEM images of DiP (a) and P2P (b). (D) AFM images of DiP (a) and P2P (b).

In this work, Di phytosomes (DiP) and P2 phytosomes (P2P) were prepared by a thin-film rehydration method (Figure 3A). Blank lipid nanoparticles without drugs (P) were also prepared with the same process. Particle size and zeta potential of the phytosomes were measured by dynamic light scattering (DLS). The characteristics of the phytosomes are summarized in Table 2. P2P showed the smallest size with an average diameter of about 53.6 ± 0.3 nm and DiP showed a slightly larger size with an average diameter of about 66.3 ± 0.3 nm (Figure 3B). P showed the maximum size with an average diameter of about 139.8 ± 1.1 nm. The results showed that the addition of Di and P2 decreased the particle size of lipid nanoparticles. Particle sizes of 100 nm diameter or less will be beneficial to the blood circulation and tumor accumulation [33]. Numerous studies have shown that cholesterol is crucial for the structural stability of liposomal membranes [34]. The existence of cholesterol analogues Di and P2 in phytosomes could improve the structural stability of phytosomes. The zeta potential values of DiP and P2P were −6.4 and −4.0 mV, respectively. Because the negatively charged particles interact weakly with negatively charged cell membranes, anionic nanoparticles could have less cytotoxicity than cationic ones [35]. In addition, it was reported that anionic nanoparticles could be inclined to interact with the lung surfactant yielding a better access into lung cells [36]. Therefore, the phytosomes we prepared with sizes below 100 nm and negative charges will be more suitable for lung cancer treatment.

**Table 2:** Characterization of different phytosomes.

no.	sample	*z*-average size/nm	PDI^a^	zeta potential/mV

1	P^b^	139.8 ± 1.1	0.227 ± 0.014	−4.6 ± 0.2
2	DiP^c^	66.3 ± 0.3	0.268 ± 0.001	−6.4 ± 1.9
3	P2P^d^	53.6 ± 0.3	0.182 ± 0.008	−4.0 ± 0.7

^a^PDI: polydispersity index; ^b^P: blank lipid nanoparticles; ^c^DiP: diosgenin phytosomes. ^d^P2P: FZU-0021-194-P2 phytosomes.

The morphology of the DiP and P2P was observed by transmission electron microscopy (TEM) and atomic force microscopy (AFM, Figure 3C,D). P2P and DiP demonstrated roughly homogeneous rod shapes in TEM but showed spherical morphology in AFM. The particle size measured by AFM/TEM was larger than the particle size measured by DLS. Because of the low zeta potential values of the prepared phytosomes, the electrostatic effects between the particles are too weak to maintain the shape. The large size measured from AFM and TEM might be attribute to the coalescence of particles during drying. According to the literature, phytosomes will display micellar shapes in aqueous solution [37]. The spherical phytosomes may probably coagulate into rods in the drying step during TEM sample preparation. From the DLS and TEM measurements, the size distribution of phytosomes seems a little broad. We used the thin-film hydration method followed by sonication to prepare the phytosomes in this study. The mean size and size distribution are significantly influenced by the sonication conditions. Further optimization of the formulation and the preparation technology are needed to improve the physicochemical properties of the phytosomes.

### Antiproliferative activity of P2P

**Figure 4:**
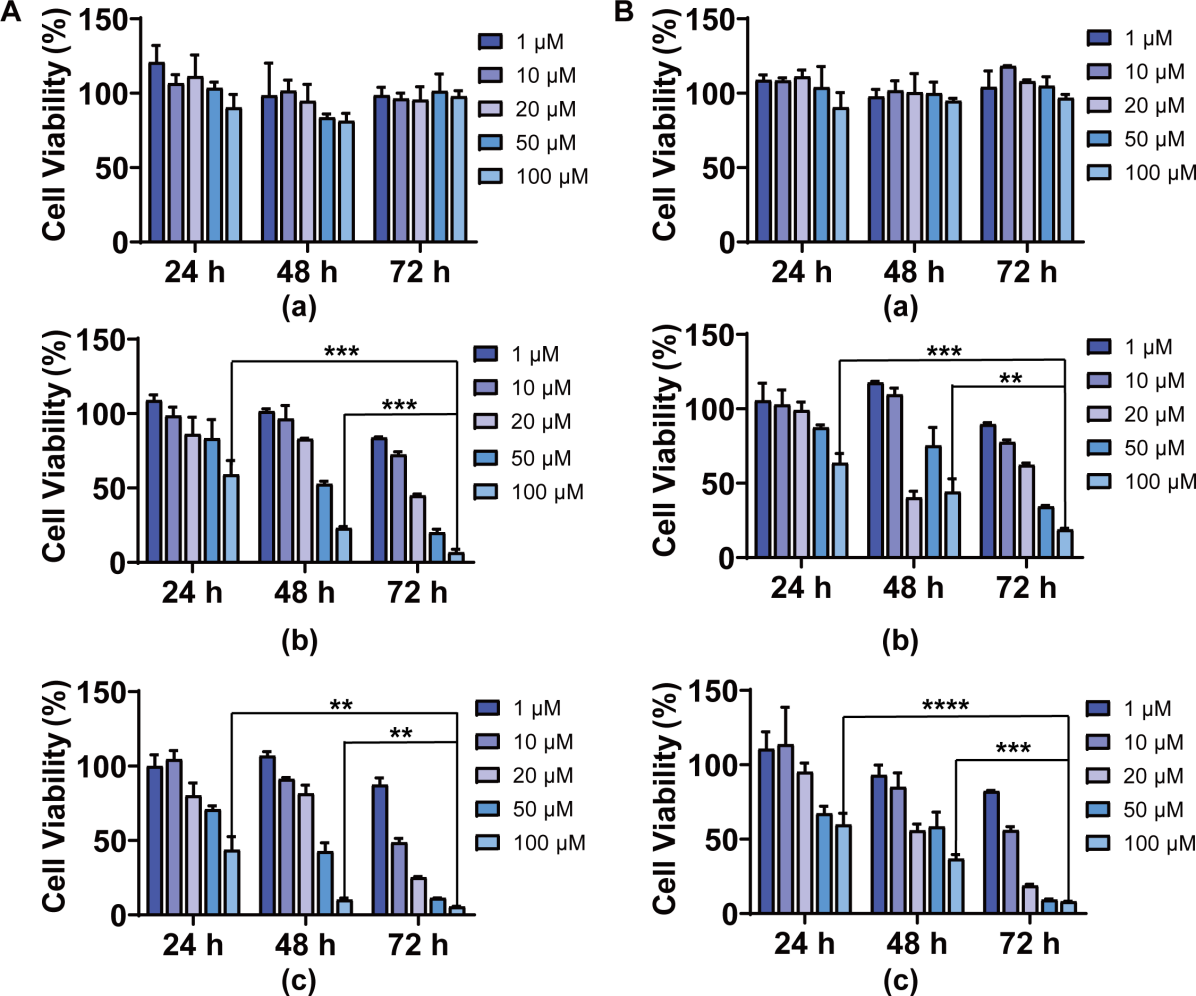
In vitro antiproliferative activity of blank lipid nanoparticle (P), DiP, and P2P. (A) The cytotoxicity of different concentrations of P (a), DiP (b), and P2P (c) against A549 cells for 24, 48 and 72 h. (B) The cytotoxicity of different concentrations of P (a), DiP (b), and P2P (c) against PC9 cells for 24, 48 and 72 h. **p < 0.01 and ***p < 0.001 compared with 24 h or 48 h treatment group by Student’s *t*-test.

To determine the cytotoxicity of DiP and P2P in lung cancer cells, cells were treated with different concentrations of P, DiP and P2P for different incubation times. The toxicity of the blank lipid nanoparticles was first investigated. As shown in Figure 4, P did not show any antiproliferative effects on A549 and PC9 cells, indicating the safety of the carrier material. DiP and P2P showed efficient antiproliferative activity in a dose- and time-dependent manner. DiP and P2P showed no obvious cytotoxicity after 24 h incubation. However, the antiproliferative effects were greatly improved when the incubation time was extended to 72 h. The results indicated that the entrapped Di and P2 could be sustainedly released from phytosomes to exert effects. As the drugs delivered to the lungs will be quickly eliminated due to large alveolar surface area, abundant capillaries and minimal transport distance, the sustained drug release delivery systems will improve the drug absorption and increase the activities [38]. Compared with DiP, P2P still showed better antiproliferative effects against A549 and PC9 cells. As comparison, the IC_50_ values of Di and P2 against A549 and PC9 cells after 72 h were also calculated (Table 1). The IC_50_ values of Di and P2 against A549 and PC9 at 72 h were lower than the IC_50_ values at 48 h. Compared with free drugs, DiP and P2P showed comparable or better antiproliferative effects after a 72 h incubation. The DiP and P2P were made up of drugs and phosphatidylcholine. The antiproliferative activities of DiP and P2P against A549 and PC9 cells originated from the drugs. Therefore, there is no huge difference in cell proliferation inhibition between cells treated with free drugs and their corresponding phytosomes. The results indicated that phytosomes could be an ideal drug delivery system for Di and its derivatives to obtain sustained release without affecting drug activity.

### In vitro anticancer mechanisms of P2P

**Figure 5:**
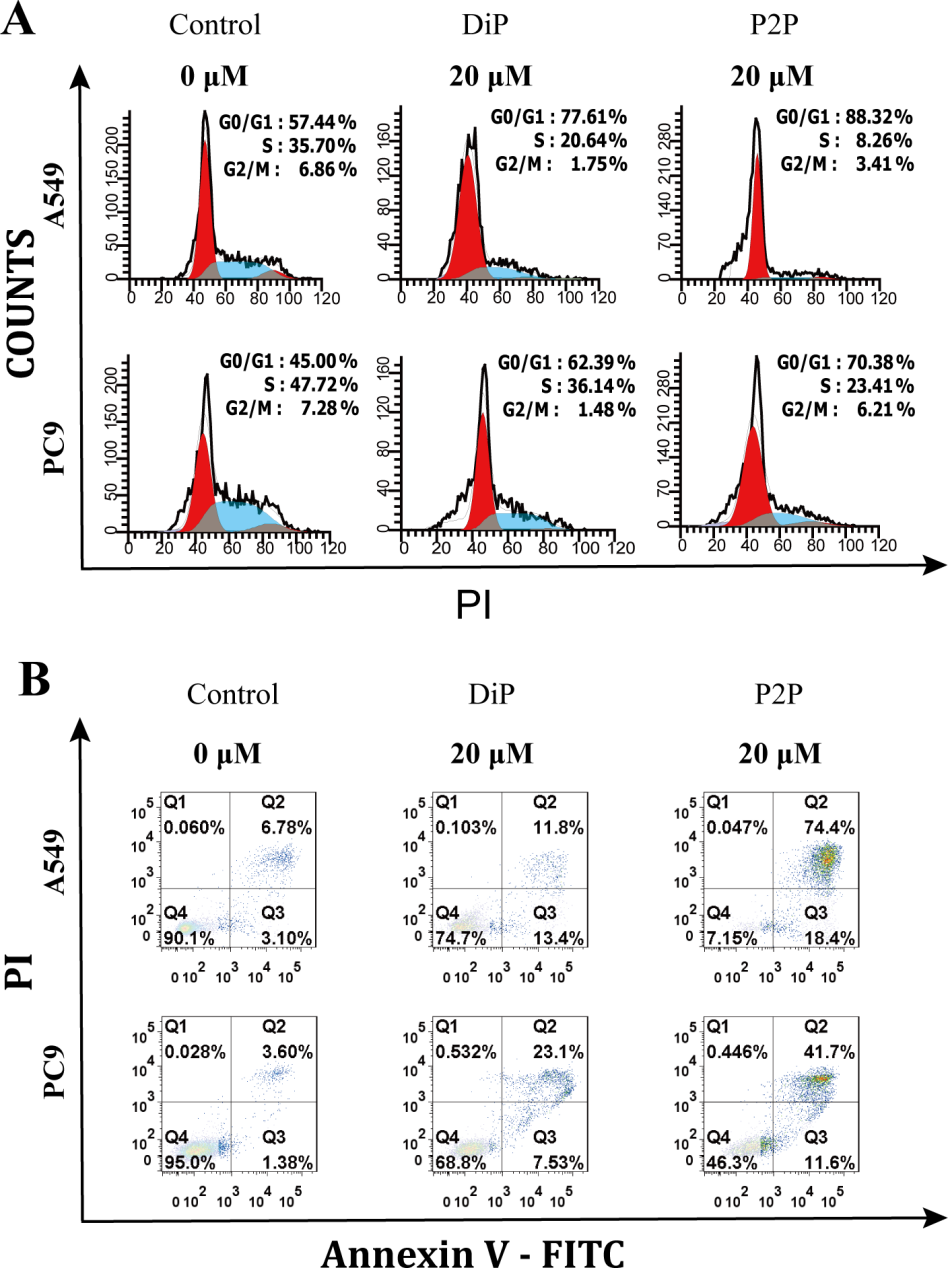
(A) Cell cycle analysis of A549 and PC9 cells after treated with DiP and P2P for 72 h. (B) Cell apoptosis analysis of A549 and PC9 cells after treated with DiP and P2P for 72 h.

Cell cycle and cell apoptosis were also investigated in A549 and PC9 cells to study the anticancer mechanisms of drug-loaded phytosomes. After 72 h incubation time, DiP and P2P induced cell cycle arrest in the G0/G1 phase in A549 and PC9 cells (Figure 5A), indicating that the actions of DiP and P2P on cell cycle regulation were the same as those of the free drugs. Compared with DiP, P2P arrested more cells in the G0/G1 phase. The results were consistent with the antiproliferation studies. After 72 h of incubation, P2P induced obvious cell apoptosis in A549 and PC9 cells (Figure 5B). The cell apoptosis induced by P2P was significantly higher than that induced by DiP, indicating that P2 loaded in phytosomes could retain its anticancer activities. As shown in Figure 2C, A549 cells treated with P2 after 48 h were almost in the early phase of apoptosis. However, A549 cells treated with P2P after 72 h were almost in the late phase of apoptosis, suggesting that the actions of P2 on induction of cell apoptosis could be altered after loading in phytosomes. One reason might be the changed drug uptake pathway. The free drugs were taken up by passive transport, while the phytosomes were taken up through endocytosis [39]. Besides, the phytosomes containing abundant phospholipids could carry amphiphilic agents to cross the cell membrane resulting in high intracellular drug concentrations, which might change the action mechanisms of P2.

## Conclusion

Di derivatives were designed and P2 was screened. P2 showed better cytotoxic activities than Di against human non-small-cell lung cancer A549 and PC9 cells. To further improve the water solubility of Di and P2, DiP and P2P were prepared with a thin-film rehydration method. DiP and P2P exhibited particle sizes less than 100 nm with oval shape and negative charges. Compared with free drugs, DiP and P2P showed comparable or better antiproliferative effects after 72 h of incubation, and P2P still showed better antiproliferative effects through inducing apoptosis and cell cycle arrest against lung cancer cells compared with DiP. All results indicated that P2P could be a promising anticancer formulation for lung cancer.

## Experimental

### Materials

3-(4,5-Dimethylthiazol-2-yl)-2,5-diphenyltetrazolium bromide (MTT), DNA-free RNaseA, and propidium iodide (PI) were purchased from Sigma-Aldrich (St Louis, USA). Di was obtained from Energy Chemical (Shanghai, China). RPMI 1640 medium and trypsin-EDTA were purchased from Gibco-BRL (Burlington, ON, Canada). Fetal bovine serum (FBS) was obtained from Gemini (Calabasas, USA). Lecithin and cholesterol were obtained from Sinopharm Chemical Reagent Co., Ltd. (China). All commercially available starting materials and solvents were reagent grade and used without further purification.

Preparative column chromatography was performed using silica gel 60 with particle size 0.063–0.200 mm (70–230 mesh, Flash). Analytical thin-layer chromatography (TLC) was carried out employing silica gel 60 F254 plates (Merck, Darmstadt). Nuclear magnetic resonance (NMR) spectra were recorded on a Bruker 400 (^1^H, 400 MHz; ^13^C, 101 MHz) spectrometer. Chemical shifts are expressed in ppm, and *J* values are given in Hz. High-resolution mass spectra (HRMS) were obtained with a Thermo Fisher Scientific Exactive Plus mass spectrometer.

### Synthesis and characterization of FZU-0021-194-P2

To a solution of Di (829 mg, 2 mmol) in EtOH (25 mL) was added FePc (28 mg, 0.05 mmol), NaBH_4_ (378 mg, 10 mmol) and KCNO (811 mg, 10 mmol). The reaction mixture was stirred at rt under air for 48 h. The solvent was then evaporated to dryness and the residue was diluted with EtOAc (20 mL) and extracted with H_2_O (20 mL). The combined organic layers were washed with saturated brine, dried over anhydrous Na_2_SO_4_, filtered, which was purified by silica gel chromatography (PE/EtOAc 4:1) to give the desired product (348 mg, 38%) as a white solid. TLC: *R*_f_ = 0.54 (PE/EtOAc 2:1). ^1^H NMR (400 MHz, CDCl_3_) δ 4.38 (d, *J* = 6.3 Hz, 1H), 4.07–3.94 (m, 1H), 3.45 (d, *J* = 10.0 Hz, 1H), 3.34 (t, *J* = 11.2 Hz, 1H), 2.04–1.94 (m, 2H), 1.87–1.80 (m, 2H), 1.76 (d, *J* = 9.9 Hz, 2H), 1.69 (d, *J* = 12.3 Hz, 2H), 1.66–1.50 (m, 7H), 1.50–1.37 (m, 6H), 1.28–1.12 (m, 6H), 1.01–0.90 (m, 6H), 0.79–0.69 (m, 6H); ^13^C NMR (101 MHz, CDCl_3_) δ 121.12, 109.32, 80.83, 67.66, 67.16, 66.92, 62.20, 55.84, 47.13, 43.67, 41.71, 40.72, 39.90, 39.39, 35.06, 34.38, 31.72, 31.70, 31.47, 30.54, 30.35, 28.89, 26.82, 21.30, 17.21, 16.61, 15.42, 14.56. HRMS (ESI) *m*/*z*: [M + H]^+^ calcd for C_28_H_43_NO_4_, 458.3265; found, 458.3280.

### Preparation of phytosomes

DiP and P2P were prepared with lecithin in the prescribed ratio (1:1, molar ratio) by the thin film hydration method. First, lecithin and Di/P2 were dissolved in chloroform at a molar ratio of 1:1. The organic solvent was evaporated using a rotary evaporator to produce a thin lipid film. Before hydration, the lipid film was dried in a vacuum drying chamber at 27 °C for 12 h. The hydrated multilamellar vesicles were sonicated by a sonicator for 20 min. Blank lipid nanoparticles (P) were also prepared with the same process without adding Di or P2.

### Characterization of phytosomes

Hydrodynamic diameter and zeta potential of the phytosomes were analyzed by DLS on a Malvern Instruments Zetasizer HS III (Malvern, UK) at room temperature. The morphology of the phytosomes was recorded by using atomic force microscopy (AFM, Multimode 8, Bruker, USA) and transmission electron microscopy (TEM, HT7700, Hitachi, Japan). The DiP and P2P were diluted with ddH_2_O to a concentration of 100 μg/mL. 2.5 μL of the dispersion was dripped on mica sheets, then washed three times with 10 μL ddH_2_O and dried by nitrogen blow before AFM observation. The AFM measurements were conducted in intelligent model and under ambient conditions with a silicon cantilever with *L* = 115 μm, *W* = 25 μm, and *T* = 605 nm. For TEM measurements, the DiP and P2P were diluted with ddH_2_O to a concentration of 10 μg/mL. 8.5 μL of the dispersion was dripped on the copper grid and dried in air overnight before imaging.

### Cell cultures

The A549 and PC9 cells were obtained from the Cell Resource Center of Shanghai Institute for Biological Sciences (Chinese Academy of Sciences, Shanghai, China). The A549 and PC9 cells were grown in normal RPMI medium containing 10% FBS. The cells were maintained in a moist cell incubator at 37 °C with 5% CO_2_.

### In vitro cell viability

Cell viability was determined by MTT assay. Cells seeded in 96-well plates at 70–80% confluence were exposed to free drugs (Di, P2) and phytosomes (P, DiP, P2P) at various concentrations for 24, 48 and 72 h. At the end of the treatment period, viability was determined by the MTT assay [40].

### Cell cycle analysis

A549 and PC9 cells were plated in six-well plates at 2 × 10^5^ cells/well. The day after plating, the cells were treated with Di or P2 for 48 h and DiP or P2P for 72 h. Then cells were trypsinized and washed with PBS. The cells were fixed with 75% pre-cooled ethanol and kept at 4 °C overnight. Fixed cells were washed with PBS and incubated with fluorescent solution (1% (v/v) Triton X-100, 0.05% PI, 0.01% RNase A) in dark for 30 min at room temperature. Finally the cells were analyzed by FACS Calibur system and the data was processed and analyzed using the ModFit software (Verity Software House, Topsham, ME) [41]. The cell amounts for each group which are analyzed by the flow cytometry are 10000 events. The representative gating for flow cytometry was shown in Supporting Information File 1, Figure S3.

### Cell apoptosis

For analysis of apoptosis, A549 and PC-9 cells were cultivated with Di or P2 for 48 h and DiP or P2P for 72 h under normoxic condition. Then, cells were harvested, washed with PBS, and collected by centrifugation. The cell suspensions were mixed with 5 μL of FITC-Annexin V, 5 μL of PI, and 500 μL of binding buffer and kept for 15 min in the dark. Subsequently, the apoptotic stages were quantitatively determined by flow cytometry [42].

### Statistical analysis

All data shown in this article were expressed as the mean ± SD for at least three separate experiments. Statistical analysis was performed using the Student's *t*-test.

## Supporting Information

File 1 Additional experimental information.

## References

[1] Li X, Li X, Li Y, Yu C, Xue W, Hu J, Li B, Wang P, Zhu F (2019). Anti-Cancer Agents Med Chem.

[2] Chen Y, Tang Y-M, Yu S-L, Han Y-W, Kou J-P, Liu B-L, Yu B-Y (2015). Chin J Nat Med.

[3] Rahmati-Yamchi M, Ghareghomi S, Haddadchi G, Milani M, Aghazadeh M, Daroushnejad H (2014). Mol Biol Rep.

[4] Bhuvanalakshmi G, Basappa, Rangappa K S, Dharmarajan A, Sethi G, Kumar A P, Warrier S (2017). Front Pharmacol.

[5] Li Y, Wang X, Cheng S, Du J, Deng Z, Zhang Y, Liu Q, Gao J, Cheng B, Ling C (2015). Oncol Rep.

[6] Tao X, Xu L, Yin L, Han X, Qi Y, Xu Y, Song S, Zhao Y, Peng J (2017). Cell Death Dis.

[7] Khosravi Z, Sedaghat R, Baluchnejadmojarad T, Roghani M (2019). Int Immunopharmacol.

[8] Feng J-F, Tang Y-N, Ji H, Xiao Z-G, Zhu L, Yi T (2017). Oxid Med Cell Longevity.

[9] Zheng L, Yin L, Xu L, Qi Y, Li H, Xu Y, Han X, Liu K, Peng J (2018). Biomed Pharmacother.

[10] Cai H, Wang Z, Zhang H-Q, Wang F-R, Yu C-X, Zhang F-X, Gao L, Zhang J, Zhao J-J (2014). Acta Pharmacol Sin.

[11] Chojnacki J E, Liu K, Saathoff J M, Zhang S (2015). Bioorg Med Chem.

[12] Masood-ur-Rahman, Mohammad Y, Fazili K M, Bhat K A, Ara T (2017). Steroids.

[13] Deng G, Zhou B, Wang J, Chen Z, Gong L, Gong Y, Wu D, Li Y, Zhang H, Yang X (2019). Eur J Med Chem.

[14] Fernández-Herrera M A, Sandoval-Ramírez J, Sánchez-Sánchez L, López-Muñoz H, Escobar-Sánchez M L (2014). Eur J Med Chem.

[15] Li F, Mei H, Xie X, Zhang H, Liu J, Lv T, Nie H, Gao Y, Jia L (2017). AAPS J.

[16] Li F, Mei H, Gao Y, Xie X, Nie H, Li T, Zhang H, Jia L (2017). Biomaterials.

[17] Mohammed A R, Weston N, Coombes A G A, Fitzgerald M, Perrie Y (2004). Int J Pharm.

[18] Janicka K, Jastrzebska I, Petelska A D (2016). J Membr Biol.

[19] Cernak T, Dykstra K D, Tyagarajan S, Vachal P, Krska S W (2016). Chem Soc Rev.

[20] White M C, Zhao J (2018). J Am Chem Soc.

[21] Chen Z, Xu J, Wu Y, Lei S, Liu H, Meng Q, Xia Z (2018). Biochem Biophys Res Commun.

[22] Das S, Dey K K, Dey G, Pal I, Majumder A, MaitiChoudhury S, kundu S C, Mandal M (2012). PLoS One.

[23] Hsieh M-J, Tsai T-L, Hsieh Y-S, Wang C-J, Chiou H-L (2013). Arch Toxicol.

[24] Pistritto G, Trisciuoglio D, Ceci C, Garufi A, D'Orazi G (2016). Aging.

[25] Nie C, Zhou J, Qin X, Shi X, Zeng Q, Liu J, Yan S, Zhang L (2016). Mol Med Rep.

[26] Zhao X, Tao X, Xu L, Yin L, Qi Y, Xu Y, Han X, Peng J (2016). Molecules.

[27] Yue X, Dai Z (2018). Curr Med Chem.

[28] Abdelkader H, Longman M, Alany R, Pierscionek B (2016). Int J Nanomed.

[29] Hou Z, Li Y, Huang Y, Zhou C, Lin J, Wang Y, Cui F, Zhou S, Jia M, Ye S (2013). Mol Pharmaceutics.

[30] Shakeri A, Sahebkar A (2016). Recent Pat Drug Delivery Formulation.

[31] Mazumder A, Dwivedi A, du Preez J L, du Plessis J (2016). Int J Pharm.

[32] Lazzeroni M, Guerrieri-Gonzaga A, Gandini S, Johansson H, Serrano D, Cazzaniga M, Aristarco V, Macis D, Mora S, Caldarella P (2017). Cancer Prev Res.

[33] Nagayasu A, Uchiyama K, Kiwada H (1999). Adv Drug Delivery Rev.

[34] Huang Z, Jaafari M R, Szoka F C (2009). Angew Chem, Int Ed.

[35] Shao X-R, Wei X-Q, Song X, Hao L-Y, Cai X-X, Zhang Z-R, Peng Q, Lin Y-F (2015). Cell Proliferation.

[36] Arick D Q, Choi Y H, Kim H C, Won Y-Y (2015). Adv Colloid Interface Sci.

[37] Semalty A, Semalty M, Rawat M S M, Franceschi F (2010). Fitoterapia.

[38] Loira-Pastoriza C, Todoroff J, Vanbever R (2014). Adv Drug Delivery Rev.

[39] Babazadeh A, Zeinali M, Hamishehkar H (2018). Curr Drug Targets.

[40] Lv T, Yu T, Fang Y, Zhang S, Jiang M, Zhang H, Zhang Y, Li Z, Chen H, Gao Y (2017). Mater Sci Eng, C.

[41] Zhang Y, Lv T, Zhang H, Xie X, Li Z, Chen H, Gao Y (2017). Biomacromolecules.

[42] Zhang H, Wu F, Li Y, Yang X, Huang J, Lv T, Zhang Y, Chen J, Chen H, Gao Y (2016). Beilstein J Nanotechnol.

