# Supplementary material for: Synthesis and potent cytotoxic activity of a novel diosgenin derivative and its phytosomes against lung cancer cells
Source: Beilstein J Nanotechnol. 2019 Sep 24;10:1933–42. doi: 10.3762/bjnano.10.189 (PMC6774070; doi:10.3762/bjnano.10.189)
Supplement: File 1 — Additional experimental information. [file Beilstein_J_Nanotechnol-10-1933-s001.pdf]

BEILSTEIN JOURNAL OF NANOTECHNOLOGY

Supporting Information

for

Synthesis and potent cytotoxic activity of a novel diosgenin derivative and its phytosomes against lung cancer cells

Liang Xu, Dekang Xu, Ziying Li, Yu Gao and Haijun Chen

Beilstein J. Nanotechnol. **2019,** *10,* 1933–1942. doi:10.3762/bjnano.10.189

Additional experimental information

License and Terms: This is a supporting information file under the terms of the Creative Commons Attribution License (http://creativecommons.org/licenses/by/4.0). Please note that the reuse, redistribution and reproduction in particular requires that the authors and source are credited.
The license is subject to the *Beilstein Journal of Nanotechnology* terms and conditions: (https://www.beilstein-journals.org/bjnano)

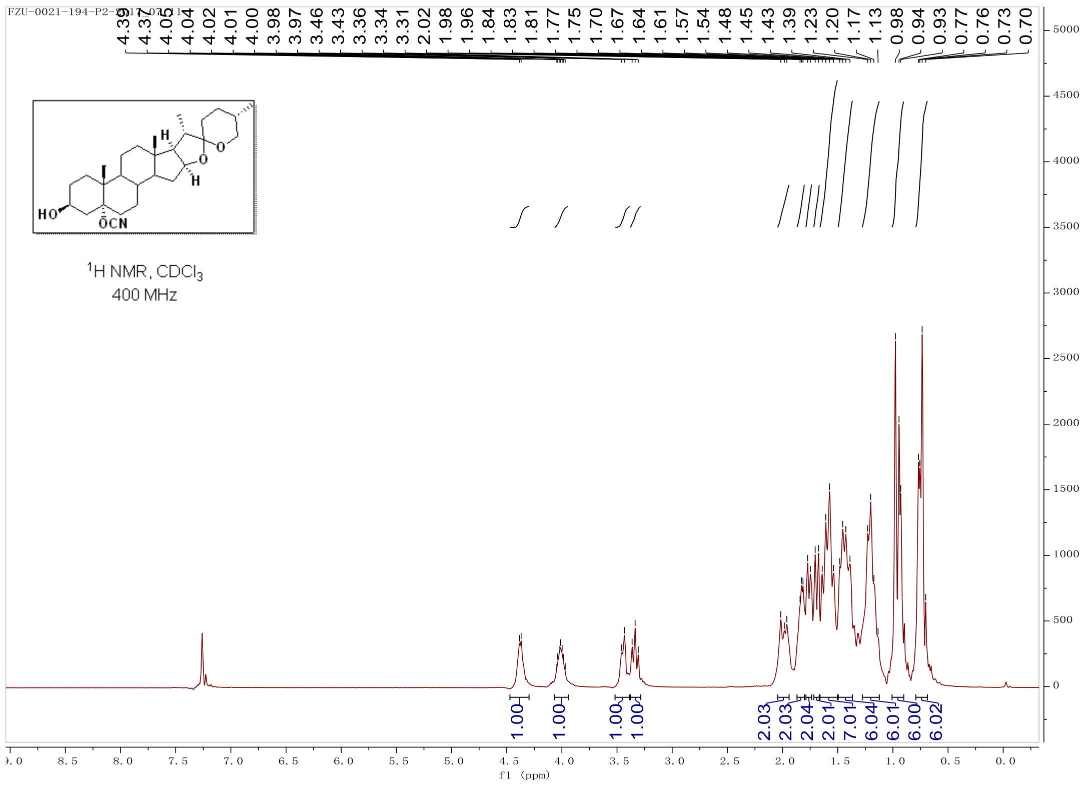

Figure S1: ^{1}H NMR spectrum of P2.

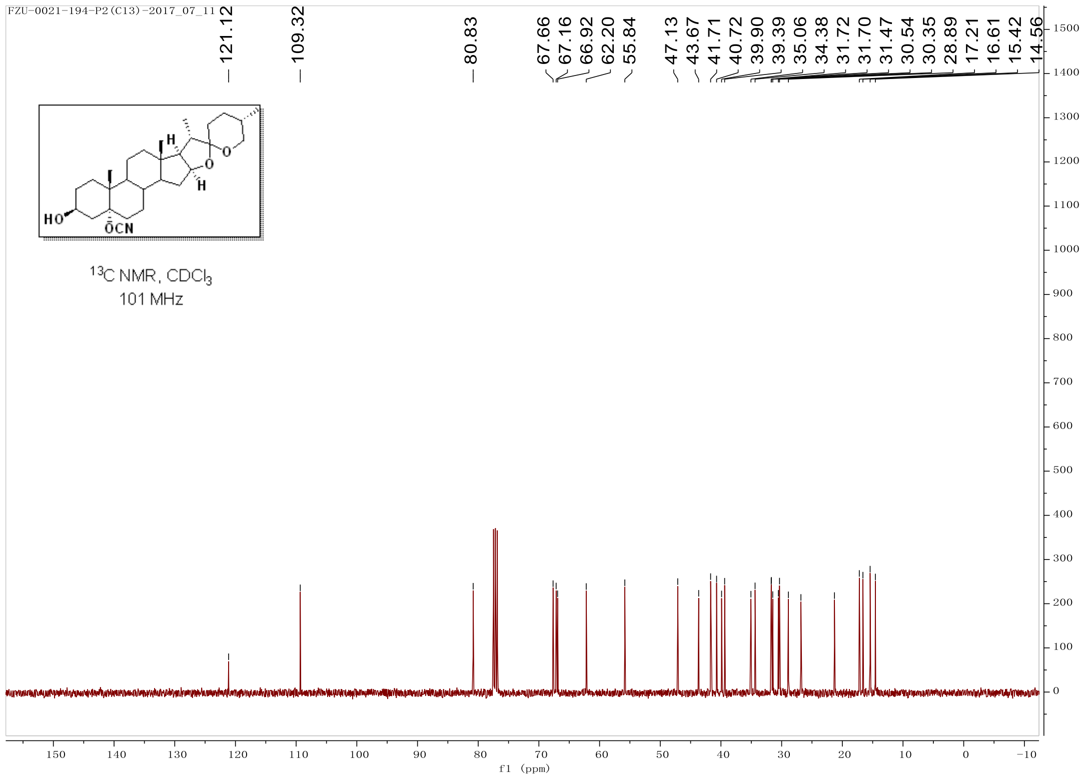

Figure S2: ^{13}C NMR spectrum of P2.

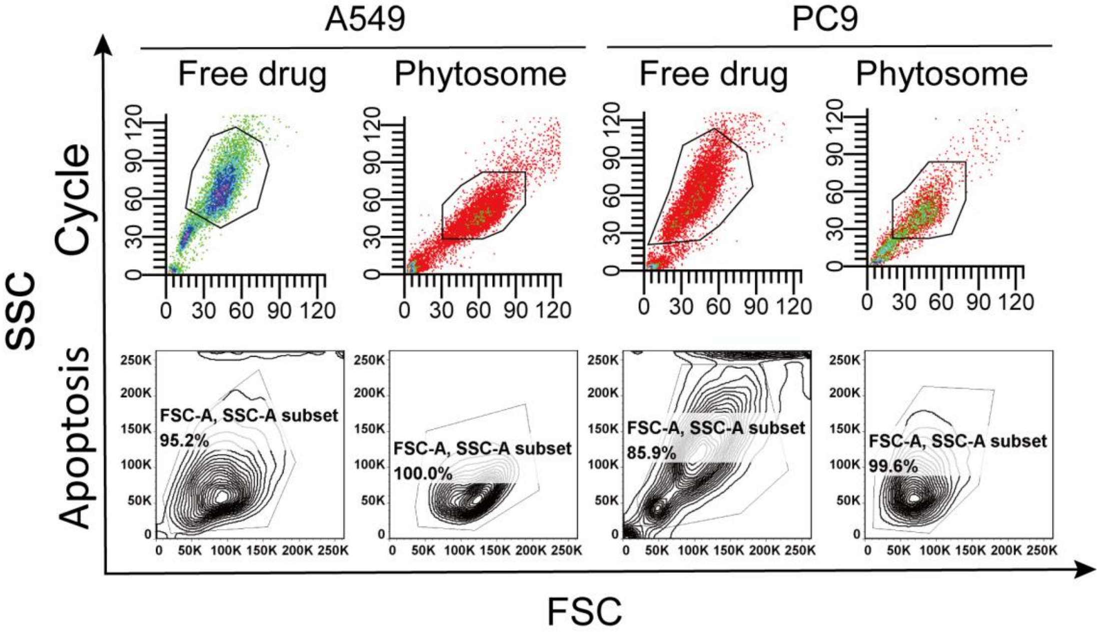

Figure S3: The gating for flow cytometry.
